# Room Temperature Phosphorescence Emission From Multi-States

**DOI:** 10.3389/fchem.2021.810458

**Published:** 2022-02-02

**Authors:** Xiaofeng Zhang, Beibei Zhang, Ji Luo, Song Guo, Chun Wei, Yongyang Gong

**Affiliations:** Guangxi Key Laboratory of Optical and Electronic Materials and Devices, College of Materials Science and Engineering, Guilin University of Technology, Guilin, China

**Keywords:** room temperature phosphorescence, solution state, organic small molecule, fluorene derivative, triplet state

## Abstract

Organic room temperature phosphorescence (RTP) materials have received considerable attention due to their fascinating photophysical properties. During the past decade, various organic luminogens exhibiting RTP emission in solid states were reported. However, the phosphorescence emission of organic compounds can hardly be observed in their solutions at room temperature. Herein, we reported two fluorene derivatives that can emit RTP in degassed organic solvents, polymer doped film, and crystalline states. Furthermore, those RTP luminogens emitted different colors with different phosphorescence lifetimes in multi-states. These results indicated that the phosphorescence performance can be adjusted flexibly in different condensed states. To our knowledge, this is the first example possessing diverse organic RTP at multi-states, including solution state.

## Introduction

Organic RTP attracts tremendous attention because of its unique characters, especially the long-lived triplet states, and exhibits immense potential applications in optoelectronic devices, information encryption, emergency exit sign, chemical sensors, bioimaging, and so on (Lin et al., 2018; Wang et al., 2019b; Gu et al., 2019; He et al., 2019; Yang J. et al., 2020; Yang Z. et al., 2020; Zhao et al., 2020; Liu R. et al., 2021; Garain et al., 2021; Zhu et al., 2021). In recent years, various strategies have been utilized to achieve RTP luminogens; for example, the introduction of heavy atom or aromatic carbonyl can effectively boost the spin-orbit coupling (SOC) constant and then facilitate the intersystem crossing (ISC) rates between the excited singlet (S_1_) and triplet states (T_n_), which is one of the most widely used strategy (Zhao et al., 2020; Zhang et al., 2021). Besides, host-guest doping (Notsuka et al., 2017; Qu et al., 2019; Lei et al., 2020; Liu X. et al., 2021; Guo et al., 2021), crystallization (Yuan et al., 2010; Gong et al., 2015; Wu et al., 2019; Nitti et al., 2020), H-aggregation (An et al., 2015; Yuan et al., 2019; Zhang L. et al., 2020; Li et al., 2021), polymer skeleton (Wang et al., 2019a; Du et al., 2019; Gao and Ma, 2021; Meng et al., 2021; Yan et al., 2021; Zhang et al., 2021), and other methods (Baroncini et al., 2017; Li et al., 2019; Wen et al., 2019), which can strengthen the rigidity of the structure, were also proposed towards the high-efficiency of RTP.

However, the most reported organic luminogens emitting phosphorescence are in solid states at room temperature or in solution state at 77 K, owing to the unstable triplet excited states, which can be easily quenched by molecular vibration and the collision with other media. Although several literature has reported efficient RTP in the common organic solvents at 77 K, these solvents are not completely liquid at 77 K. Thus, it is extremely difficult and of great attractiveness to exploit novel purely organic single molecular phosphorescence not only in the solid state but also in the solvent state at room temperature. In 1978, Turro et al. reported phosphorescence of naphthalene and triphenylene in purified and degassed solutions of 1, 2-dibromoethane. In addition, 1, 4-dibromonaphthalene in common solvent acetonitrile (nitrogen purged) can exhibit obvious phosphorescence at room temperature (Turro et al., 1978). In 2018, George et al. reported a supramolecular assembly using inorganic layered silicate and bromine substituted naphthalene derivatives, which showed bright phosphorescence in dilute aqueous solution and amorphous films under ambient conditions. Liu group reported enhancement ultralong bright RTP in solution by host-guest complexation interaction between phosphors and cucurbit[6, 7, 8]urils (Zhang Z. Y. et al., 2020).

Most recently, various luminogens exhibiting ambient phosphorescence in the aqueous phase were reported. Hisaeda et al. reported naphthalenediimide halobenzoate triad molecules, which exhibited evident red RTP in the suspended aqueous solution (Ono et al., 2021). However, because of the hydrophobicity of these molecules, the solution was not true solution. Ma group developed a flexible porous water-soluble supramolecular organic framework, which could efficiently exhibit phosphorescence in both the aqueous phase and film state at room temperature (Xu et al., 2021). Yang group reported a water-resistant carbon dot with a high phosphorescent quantum yield of 23% in aqueous environment and this material was successfully applied for the fingerprint recognition and advanced information encryption (Su et al., 2021). Su et al. prepared a kind of carbon dot composites using a molten salt method, which exhibited bright RTP with a quantum yield of 26.4% and a lifetime of 1.28 s. In particular, their aqueous dispersion showed obvious RTP behaviors, and then these phosphors were utilized to detect temperature and pH in the aqueous phase (Su et al., 2021). Liang et al. reported a kind of silica capsulated water-soluble carbon nanodots with the lifetime and phosphorescence quantum yield of 1.86 s and 11.6%, respectively. These features allowed them to apply for afterglow imaging *in vivo*/*in vitro* (Liang et al., 2020). In spite of extensive efforts which have been contributed to developing RTP in both solvent and solid state, including crystal and amorphousness, up to now, very few species, especially purely organic molecule, can achieve the requirements, let alone the effective regulation of the photophysical properties in different states.

Fluorene and its derivatives are a kind of very important organic chromophores with π conjugation, which have been often used in organic semiconductor devices (Xie et al., 2012). Rational modification of fluorene derivatives can achieve extraordinary photophysical properties [delayed fluorescence (Liang et al., 2018; Liu et al., 2020; Zhu et al., 2020), RTP (Bruzzone and Badía, 1990; Wang et al., 2018; Chen et al., 2021), mechanochromism (Matsuo et al., 2019; Tan et al., 2020), and so forth]. In 2013, Takeuchi et al. reported a fluorene derivative substituted by bromo and formyl groups, exhibiting distinct phosphorescence in some common organic solvents at room temperature with the phosphorescence quantum yield of 5.9% in chloroform at 298 K under N_2_ atmosphere (Xu et al., 2013). However, this derivative was a colorless viscous liquid at room temperature (Vijayakumar et al., 2011).

Herein, two traditional and frequently used intermediates of photoelectric materials, 2-bromo-9,10-diphenylfluorene (**BDF**) and 2,7-dibromo-9,10-diphenylfluorene (**DBDF**), were carefully selected to investigate their phosphorescent properties. In these two compounds, the twisted structure and large steric hindrance effect of 9,10-diphenylfluorene can suppress the detrimental π-π stacking and reinforce the rigidity of the molecules. In addition, the modified bromine atom can promote the ISC process and increase SOC constant on account of the heavy-atom effect. Interestingly, two compounds exhibited obvious RTP not merely in crystalline state and amorphous film, but also in the solution state. Impressively, emitter **BDF** showed different maximum phosphorescent emission peaks at different states. In brief, the generation and efficacious adjustment of triplet energy level at multi-states, especially the solution state, was achieved successfully. To our knowledge, this is the first purely organic molecule possessing diverse RTP at multi-states, including solution state.

## Results and Discussion

**FIGURE 1:**
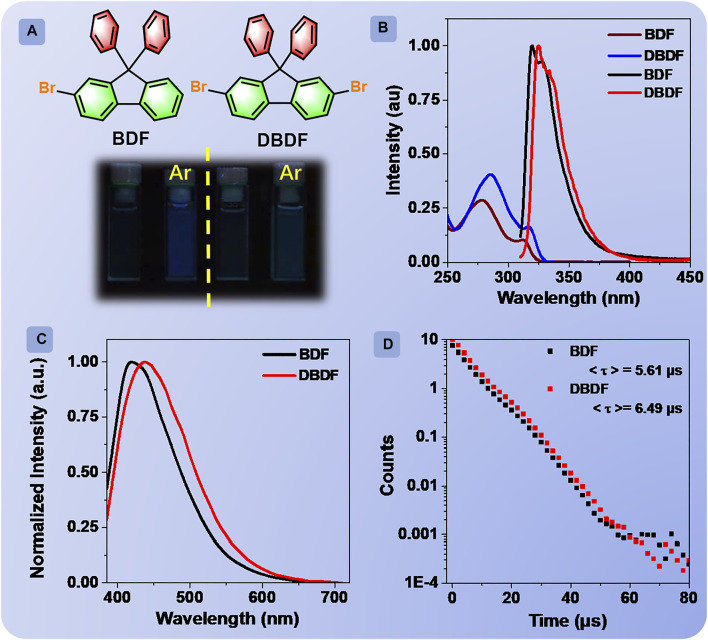
Chemical structures of **BDF** and **DBDF (A)**; absorption (blue and brown line) and emission (black and red line) spectra of **BDF** and **DBDF** in distilled chloroform solution **(B)**; emission spectra of **BDF** and **DBDF** in Ar degassed chloroform solution **(C)**; phosphorescence decay curves of **BDF** and **DBDF** in Ar degassed chloroform solution **(D)**; luminescent photographs of **BDF** (left) and **DBDF** (right) in chloroform and Ar degassed chloroform solutions under irradiation at 365 nm UV light, concentration: 2.0 × 10^−5^ M **(A)**.

Compounds **BDF** and **DBDF** (Figure 1A) were purchased commercially and purified by recrystallization. Then, the chemical structures were fully characterized and confirmed by nuclear magnetic resonance (NMR) spectroscopy, high-performance liquid chromatography (HPLC), thin-layer chromatography (TLC), and X-ray crystallography (CCDC: 2109386-2109387) (Supplementary Figures S1–S9; Supplementary Table S1).

The absorption spectra of **BDF** and **DBDF** in chloroform at the concentration of 2 × 10^−5^ M exhibited the character of spin-allowed π-π* transition with the maximal peak at 312 and 317 nm (Figure 1), respectively. Then, the emission behaviors of **BDF** and **DBDF** in chloroform solutions (2.0 × 10^−5^ M) at room temperature were investigated. The prompt (FL) spectra were carried out as shown in Figure 1B. The emission cannot be observed with the naked eye for the two solutions when excited at 290 nm under air atmosphere. However, the FL spectra for **BDF** and **DBDF** showed one peak at 320 and 325 nm, with the corresponding quantum efficiency of 0.54 and 0.64%, respectively, assigned to the ultraviolet fluorescence unambiguously. Then, the FL spectra of **BDF** and **DBDF** in different common solvents under air atmosphere were measured in detail. As shown in Supplementary Figure S10, the peak profile is almost corresponding to that of the chloroform solution. Subsequently, the FL behaviors of their solutions bubbled argon gas (Ar) for 30 min were studied. **BDF** and **DBDF** in chloroform solution deaerated with Ar showed bright blue and green emission color, respectively, when excited at 365 nm as shown in Figure 1A. Delayed emission (PL) spectra (delay time, *t*_*d*_ = 0.1 ms) of **BDF** and **DBDF** (Supplementary Figure S11) peaked at 416 and 440 nm with the corresponding lifetimes/quantum efficiency of 5.61 μs/1.51% and 6.49 μs/1.32%, respectively. This phenomenon cannot be observed under air conditions, which can be ascribed to the phosphorescent emission without doubt. It is important to note that there exist very rare organic compounds emitting phosphorescence in solution at room temperature (Shu et al., 2020). The excitation spectra of two compounds for the phosphorescent peak showed that the optimal excitation wavelengths for **BDF** and **DBDF** were both 365 nm as shown in Supplementary Figure S12. In addition, the phosphorescent intensity of the two compounds at the maximum emission peak is proportional to the concentration of the solutions (Supplementary Figure S13), suggesting that the phosphorescent emission originates from the monomeric species and no molecular interactions existed in solution. Besides, the onset of the phosphorescence spectra of BDF and DBDF can be observed in Figure 1C, peaking at 660 and 675 nm, respectively. The onset of the fluorescence spectra of BDF and DBDF can be observed in Figure 1B, peaking at 440 and 425 nm, respectively. Then, ΔE_ST_ of BDF and DBDF can be calculated as 0.94 and 1.08 eV, respectively.

**FIGURE 2:**
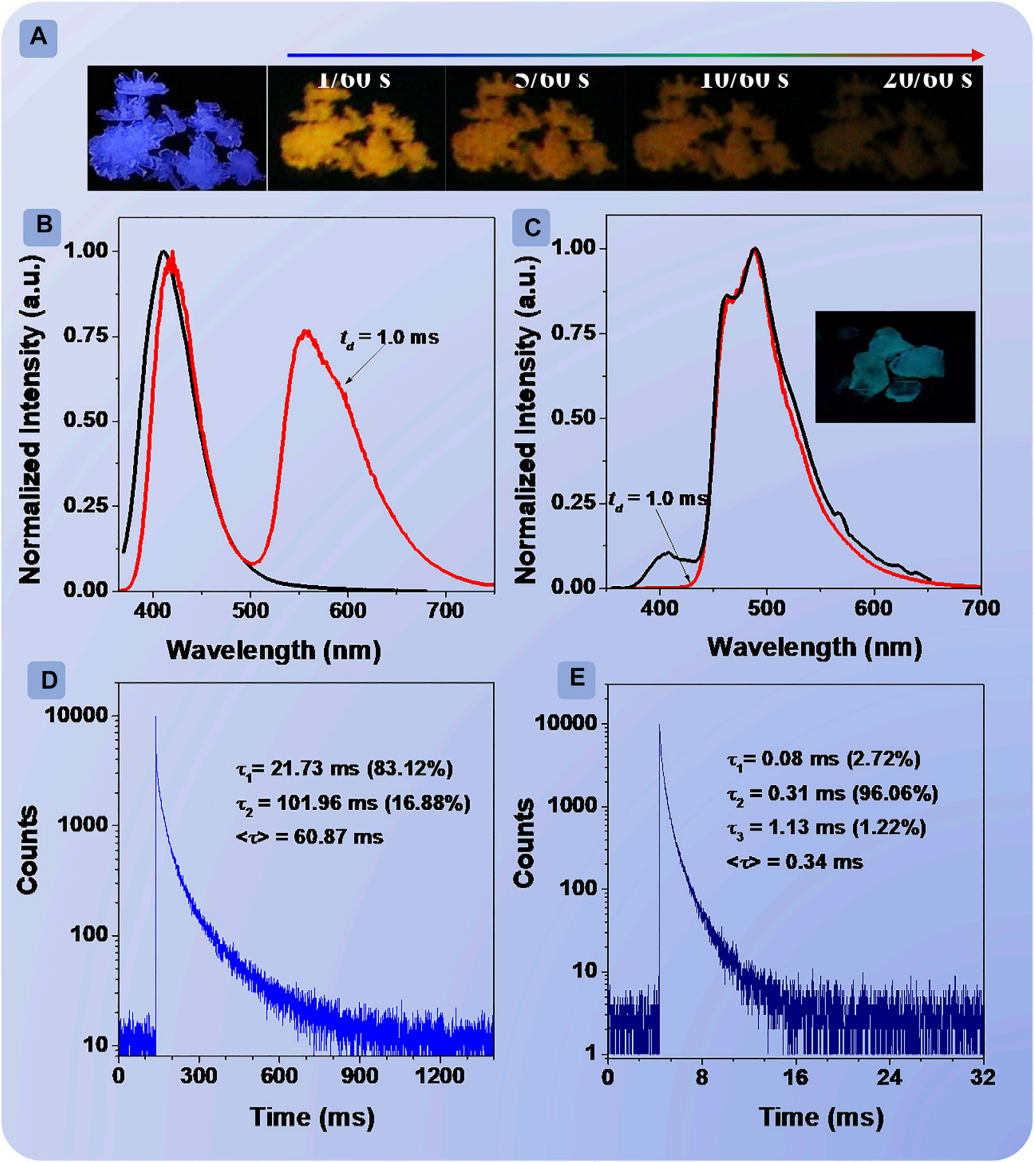
Photographs of **BDF** crystals under irradiation of 312 nm UV light at different delay times after turning off the UV lamp **(A)**; prompt (black line) and delay (red line) spectra of **BDF (B)** and **DBDF (C)**, delay time = 1.0 ms; phosphorescence decay curves of crystalline powders of **BDF (D)** and **DBDF (E)** monitored at 556 and 489 nm, respectively.

Then, the properties of the excited state in crystals were studied systematically. The XRD patterns of **BDF** and **DBDF** exhibited evident diffraction peaks as shown in Supplementary Figure S14, confirming the excellent crystallinity. **BDF** and **DBDF** showed blue (Figure 2A) and green colors under 312 nm UV lamp, respectively. The FL spectrum of **BDF** (Figure 2B, black line) peaked at 411 nm with the lifetime/quantum efficiency of 0.81 ns/7.93% (Supplementary Figure S15, black line), and **DBDF** (Figure 2C, black line) showed a weak peak at 408 nm (0.66 ns/1.38%) (Supplementary Figure S15, red line) and an intense peak at 491 nm. After ceasing the 365 nm UV lamp, **BDF** exhibited orange phosphorescence that could be observed with the naked eye (Figure 2A). However, no obvious emission can be observed from **DBDF** (Figure 2C). Next, the PL spectrum (*t*_*d*_ = 1.0 ms) of **BDF** was carried out (Figure 2B, red line), which exhibited two peaks at 417 and 556 nm, respectively. The peak at 417 nm with a lifetime of 5.72 μs was similar to the prompt spectra of **BDF**. The temperature-dependent lifetime experiment of BDF crystal at 417 nm was carried out as shown in Supplementary Figure S16. Overall, results showed that the lifetime declined accompanied by an increase in temperature, which may be assigned to the delayed fluorescent (DF). Meanwhile, the peak at 556 nm was ascribed to the phosphorescent emission with a lifetime of 60.87 ms. Besides, the PL spectra (*t*_*d*_ = 1.0 ms) of **DBDF** (Figure 2C, red line), similar to its FL spectra, showed one peak at 489 nm with a lifetime of 0.34 ms, indicating that the prompt emission of **DBDF** was mainly composed of the phosphorescent emission. Such a relatively short lifetime resulted in no obvious afterglow visible to the naked eye for **DBDF**. Compared with **BDF**, it was unexpected that the introduction of an extra bromine atom had a great impact on the phosphorescence behaviors.

Furthermore, the FL and PL spectra of **BDF** and **DBDF** at crystalline state under various temperatures were investigated as shown in Supplementary Figure S16. The FL and PL spectra of **BDF** shared similar outline peaking at 412 and 508 nm at 78 K, at the excitation of 300 nm under vacuum. The intensity of the peak at 412 nm assigned to DF emission increased when the temperature raised to 150 K from 78 K. Then, it gradually decreased with the increase in temperature due to the violent vibration and collision of the excitons. Impressively, the intensity of phosphorescent emission decreased dramatically as the temperature increased to 400 K from 78 K, accompanied by a great redshift of the wavelength from 508 to 570 nm. Meanwhile, the intensity is in inverse proportion to the increase in temperature. As for compound **DBDF**, the phosphorescent emission also decreased along with the increase in temperature. Interestingly, the phosphorescent wavelength showed a blueshift from 507 to 461/487 nm when the temperature increased to about 200 K from 78 K. This phenomenon may result from the destruction of the balance between conjugation effect and inductive effect of the halogen atom bromine as the variation of the temperature.

To gain further insights into the phosphorescent mechanism in solution and crystalline state, crystal structures of the **BDF** and **DBDF** were studied as shown in Supplementary Figures S8, S9 and Supplementary Table S1. In the crystalline state, they both exhibited C-H⋅⋅⋅π (2.859 Å) interaction. In addition, **BDF** possessed C-H⋅⋅⋅H-C (2.358 Å) interaction, and **DBDF** showed numerous Br⋅⋅⋅π interactions with a distance from 3.394 to 3.541 Å. These short contacts can effectively enhance the rigidity of the structure and decrease the non-radiative transition of the triplet state excitons, thereby boosting the ISC process, increasing the SOC constant, and then promoting the phosphorescent emission. Additionally, the dihedral angles between two benzene rings connected by the sp^3^ carbon at 9/10 position of fluorine for **BDF** and **DBDF** were 114.71° and 111.83°, respectively, which can avoid the π-π stacking.

**FIGURE 3:**
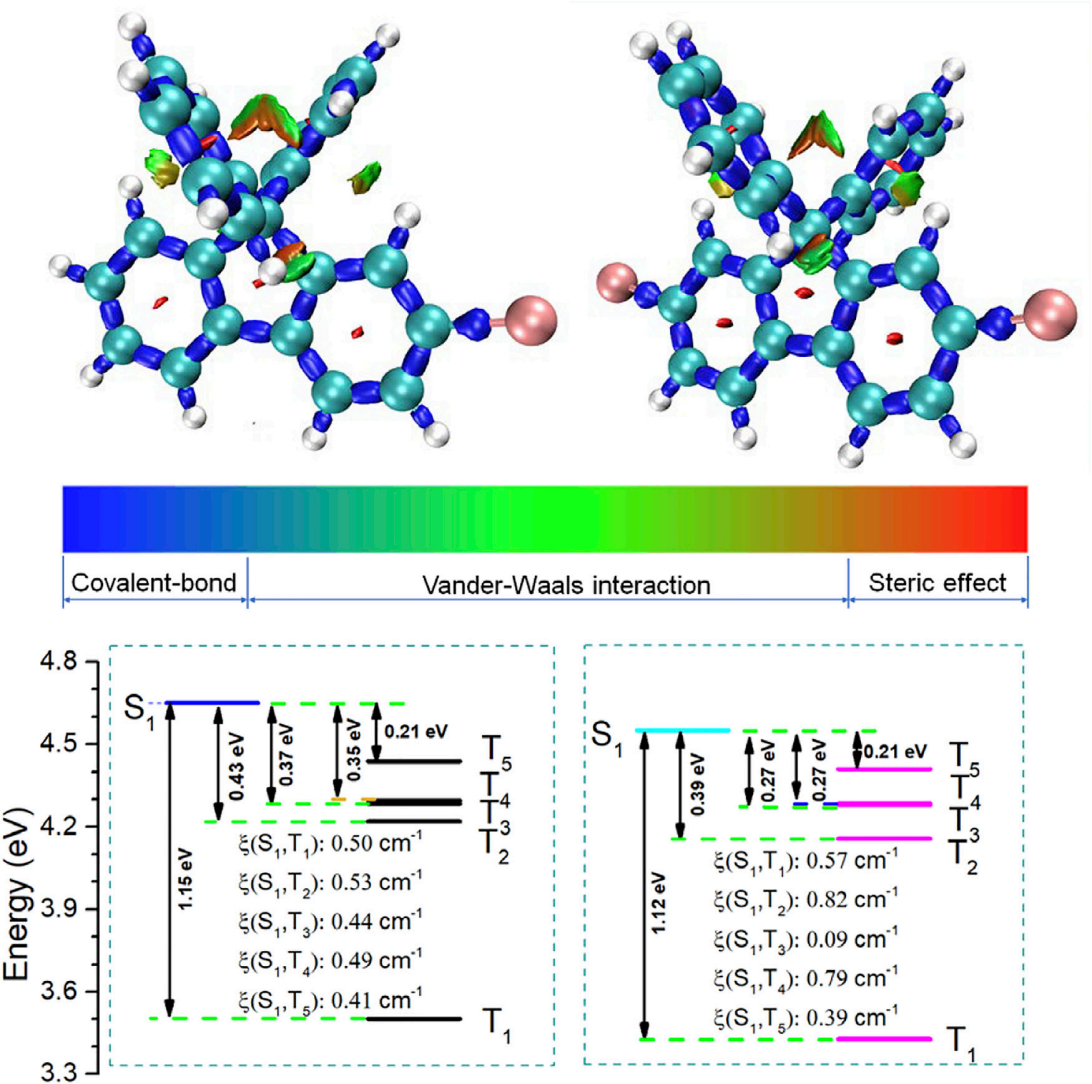
Isosurface maps of IRI = 1.0; the grid spacing for IRI is 0.12 Bohr; and red, green, and blue colors mean steric hindrance, van der Waals force, and covalent bond intramolecular, respectively (top). The energy level diagrams of **BDF** and **DBDF** (S_0_ = 0 eV). SOC: ξ (S_1_–T_n_) (cm^−1^) (bottom).

In order to further understand the RTP behaviors, theory calculation was conducted using a recently reported method, a new real space function named interaction region indicator (IRI) (Lu and Chen, 2021), which can visually reveal chemical bonding and weak interaction regions by Multiwfn (Lu and Chen, 2012). As shown in Figure 3, the IRI analysis results indicated that steric hindrance and van der Waals force restricted the free rotation of the benzene ring at the 9 and 10 positions of fluorene. Therefore, in addition to the heavy atom effect of Br atoms, the RTP performance in solution is also closely related to the steric hindrance, which was disadvantageous to the emission via the generation of excimers, and it may be responsible for the effective RTP in solutions. Moreover, the SOC constants and energy gap (Eg) between single and triplet states in the gas state calculated by the time-dependent density functional theory (TD-DFT) are shown in Figure 3 (Supplementary Figure S17; Supplementary Tables S2–S4). According to the Franck–Condon principle, the ISC rate is mainly determined by the *E*_g_ and SOC constants between excited singlet and triplet states (Gong et al., 2015). Small energy gaps and large SOC constants are favorable for the ISC process. There are five excited triplet states (T_1_ ∼ T_5_) lying below the first excited singlet state (S_1_), suggesting the possible occurrence of ISC process of S_1_ ↔ T_1_, S_1_ ↔ T_2_, S_1_ ↔ T_3_, S_1_ ↔ T_4_, S_1_ ↔ T_5_. Taking compound **BDF** for example, the SOC constants and the corresponding *E*_g_ of the **BDF** are S_1_–T_1_ (0.50 cm^−1^, 1.15 eV) and S_1_–T_5_ (0.41 cm^−1^,0.21 eV), respectively. Appropriate SOC constant and low energy level gap in S_1_-T_n_ made the ISC process possible to achieve RTP emission. These calculation results were in good agreement with the experimental results.

**FIGURE 4:**
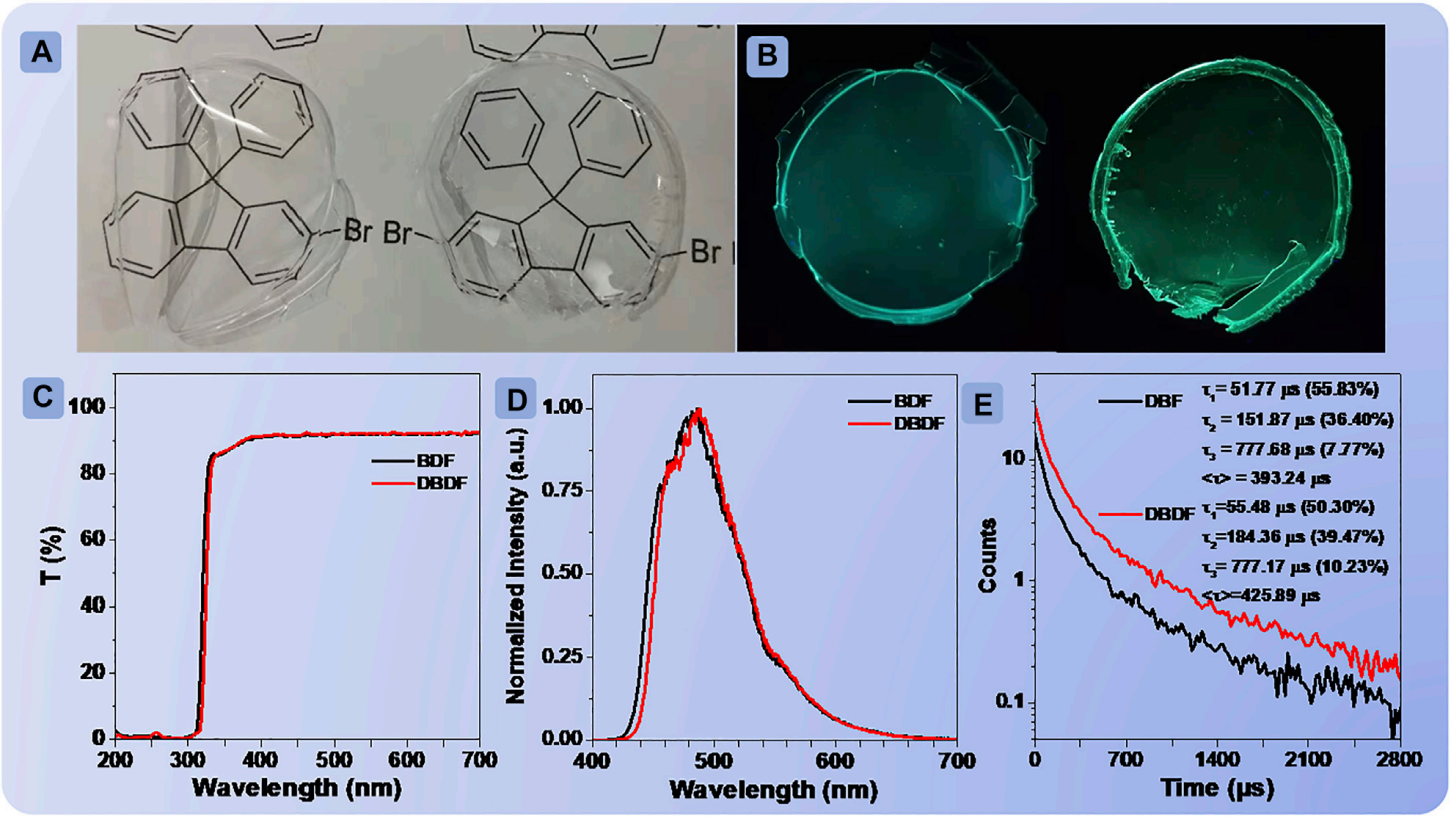
Photographs of **BDF** and **DBDF (A)** doped into PMMA films under sunlight; transmittance curves of the PMMA films doped with **BDF** and **DBDF (C)**; photographs of PMMA films doped with **BDF** and **DBDF (B)** under irradiation at 312 nm UV light; phosphorescence emission spectra **(D)** and lifetimes **(E)** of the two films at room temperature.

Then, the films fabricated by doping the phosphors into polymethyl methacrylate (PMMA) with a mass ratio of 1:100 were presented to investigate the monomolecular phosphorescent performance. The photographs of the films are shown in Figure 4A. It can be seen that the films did not block the information behind them. Subsequently, the transmittance spectra of the films were measured, as shown in Figure 4E. The results showed that few percentages of the light could be absorbed, and they exhibited excellent transmittance (≥87%) from the visible light regions of 336–700 nm. The XRD patterns of the films (Supplementary Figure S18) were recorded and showed no evident diffraction peaks, confirming the amorphous state. The fluorescent and phosphorescent behaviors of the two films were carried out at ambient conditions. Interestingly, the FL/PL spectra and phosphorescence lifetime of **BDF** and **DBDF** both exhibited similar emission performance as shown in Figures 4D,E and Supplementary Figure S19, peaking at 480/480 and 490/490 nm, with the corresponding lifetimes/quantum efficiency of 393.24 ms/4.96% and 425.89 ms/6.31%, respectively. The low-temperature FL/PL spectra of the two compounds at 78 K showed the same profile but with fine structure (Supplementary Figure S20). On the one hand, the polymer matrix restricts intramolecular rotation and enhances the radiation transition. On the other hand, PMMA doped film can avoid the invasion of water and oxygen to the matrix effectively. This is similar to the previously reported RTP emission from small molecules that doped polymer films (Cheng et al., 2018; Garain et al., 2021).

## Conclusion

In summary, in this work, we reported two fluorene derivatives that can emit RTP in degassed organic solvents: polymer doped film and crystalline states. Furthermore, compound **BDF** emitted blue, green, and yellow RTP in solution, film, and crystal states. The phosphorescence lifetime at room temperature ranges from microseconds to milliseconds. These results indicate that the emission color and lifetime of RTP can be adjusted flexibly by different condensed states. In addition to the heavy atom effect of Br, the intra-molecular steric effect and van der Waals force, which can restrict intramolecular rotation, played a vital role in the phosphorescence emission of the solution. To our knowledge, this is the first example of organic RTP at multi-states, including solution. The present organic RTP emission from multi-states would be beneficial for the exploitation of novel RTP luminogens and capable of being applied in the fields of organic optoelectronic devices, biosensors, data storage, and so forth.

## Data Availability

The original contributions presented in the study are included in the article/Supplementary Material. Further inquiries can be directed to the corresponding authors.
