# Supplementary material for: Room Temperature Phosphorescence Emission From Multi-States
Source: Front Chem. 2022 Feb 2;9:810458. doi: 10.3389/fchem.2021.810458 (PMC8847601; doi:10.3389/fchem.2021.810458)
Supplement: Supplementary file 1 [file DataSheet1.PDF]

Supplementary Material

Room temperature phosphorescence emission from multi-states

Xiaofeng Zhang, Beibei Zhang, Ji Luo, Song Guo*, Chun Wei and Yongyang Gong*

Guangxi Key Laboratory of Optical and Electronic Materials and Devices, College of Materials Science and Engineering, Guilin University of Technology, No.12 Jian'gan Rd., Qixing District, Guilin 541004, China. E-mail: 529801643@qq.com (S.G.), 1986024@glut.edu.cn (C.W.), yygong@glut.edu.cn (Y.G.)

Experimental Section

Instruments.

^{1}H NMR (400 MHz) and ^{13}C NMR (100 MHz) spectra were recorded on a Bruker AVANCE 400 NMR spectrometer in deuterated solvent at room temperature, and chemical shifts were reported in ppm relative to tetramethylsilane (TMS, δ = 0 ppm). XRD measurements were conducted on powders with a X'Pert PRO diffractometer (PANalytical, Holland). High performance liquid chromatography (HPLC) was carried out by SHIMADZU LC-20A with CH_3CN solution as the running buffer. Absorption and measurements were performed on a UV3600 UV-Vis spectrophotometer. The emission spectra, solids quantum yields and lifetime were recorded on a FluoroMax-4 fluorescence spectrophotometer. Quantum yields (Φ) of solution were estimated using 2-Aminopyridine (Φ = 60% in 0.1 N H_2SO_4) as standard, while solid-state efficiencies were determined by an integrating sphere.

Materials.

2-bromo-9,10-diphenylfluorene (BDF), 2,7-dibromo-9,10-diphenylfluorene (DBDF) and methacrylic acid methyl ester polymer (PMMA) were purchased from Adamas Reagent Ltd. Trichloromethane (TCM) was distilled under normal pressure from calcium hydride under nitrogen immediately prior to use.

Computational Methods

Geometry optimizations and frequency calculations were performed by using density functional theory (DFT) for the ground (S_0) state and time-dependent DFT (TD-DFT) for the lowest excited singlet (S_1) state with the M06-2X functional and def-TZVP basis set. It is known that M06-2X is an ideal choice for main group elements. All above calculations were carried out with the Gaussian 16 B.01 package. Gaussian calculation results are analyzed by

Multiwfn and VMD software. Besides, the spin-orbit coupling constant and vertical excitation energy of singlets and triplets were calculated at M06-2X/def-TZVP level with ORCA 4.2 software.

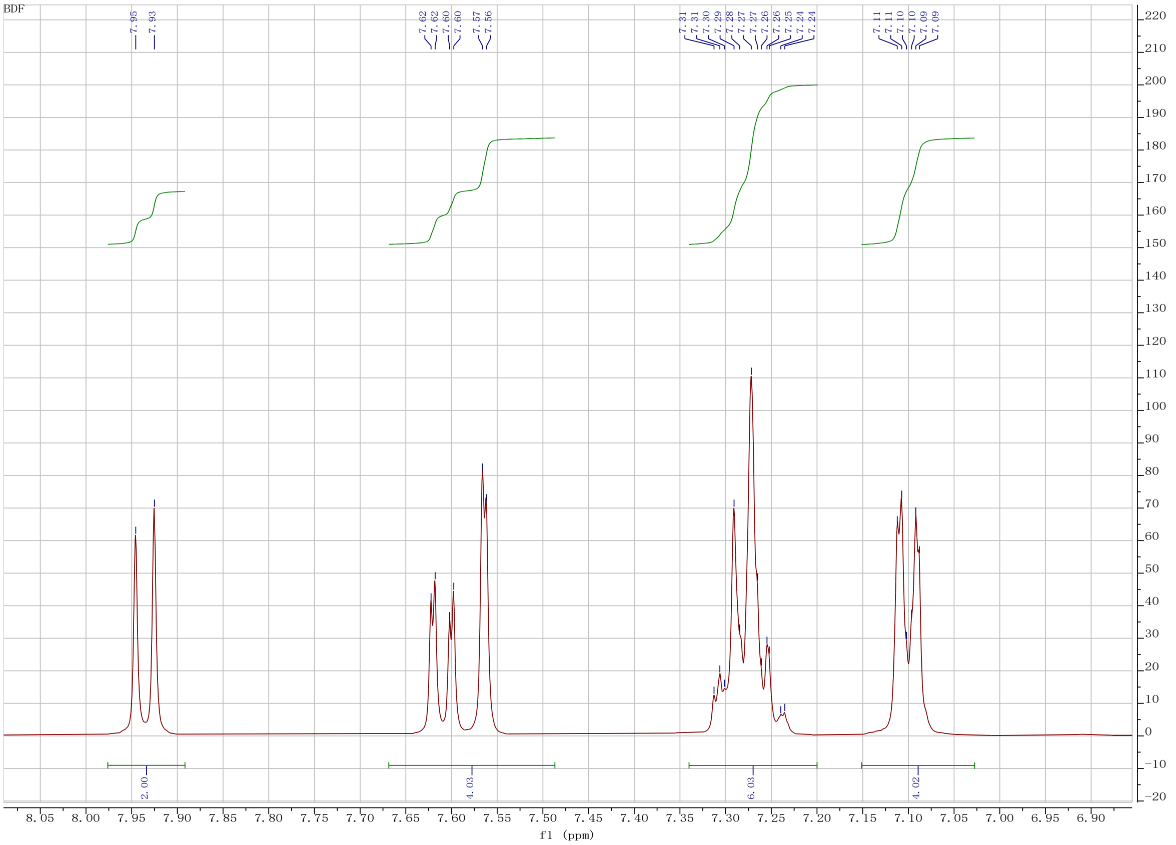

Figure S1 ^{1}H NMR spectrum of BDF. ^{1}H NMR (400 MHz, DMSO-d_6) δ 7.93 (d, J = 8.0 Hz, 2H), 7.55 – 7.67 (m, 4H), 7.20 – 7.35 (m, 6H), 7.05 – 7.16 (m, 4H).

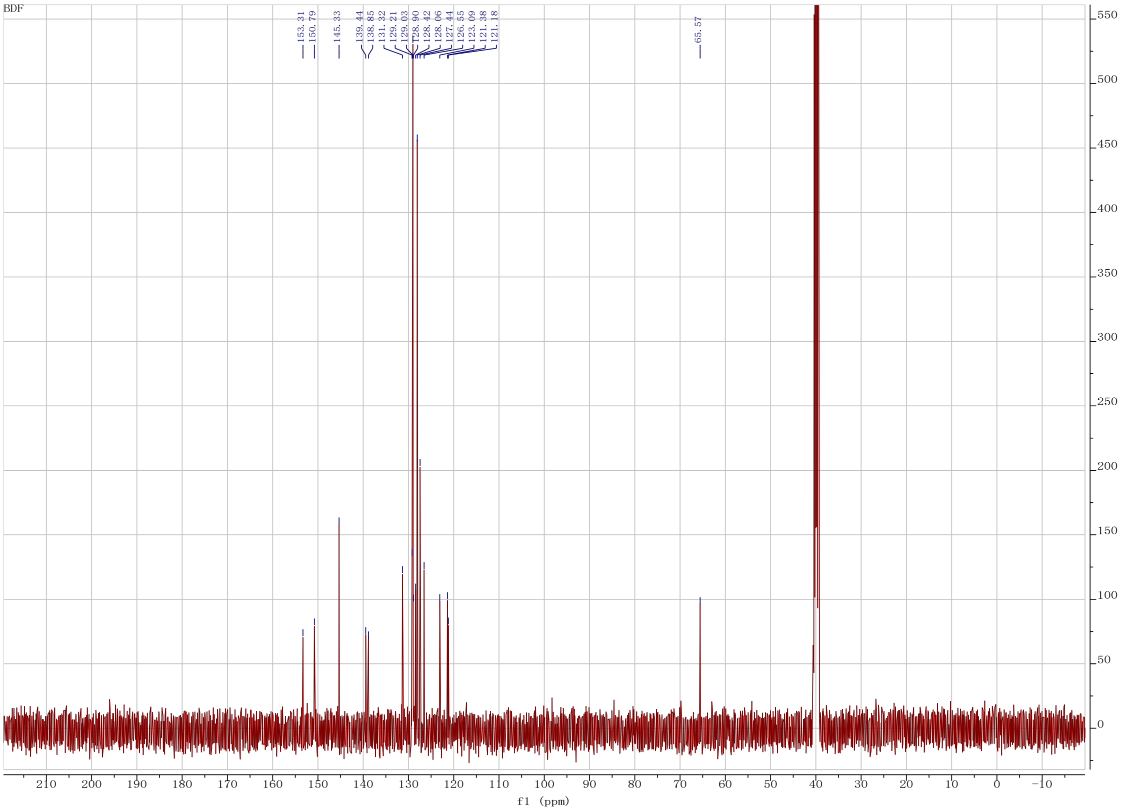

Fig. S2 ^{13}C NMR spectrum of BDF, ^{13}C NMR (100 MHz, DMSO) δ 153.31, 150.79, 145.33, 139.44, 138.85, 131.32, 129.21, 129.03, 128.90, 128.42, 128.06, 127.44, 126.55, 123.09, 121.38, 121.18, 65.57.

.

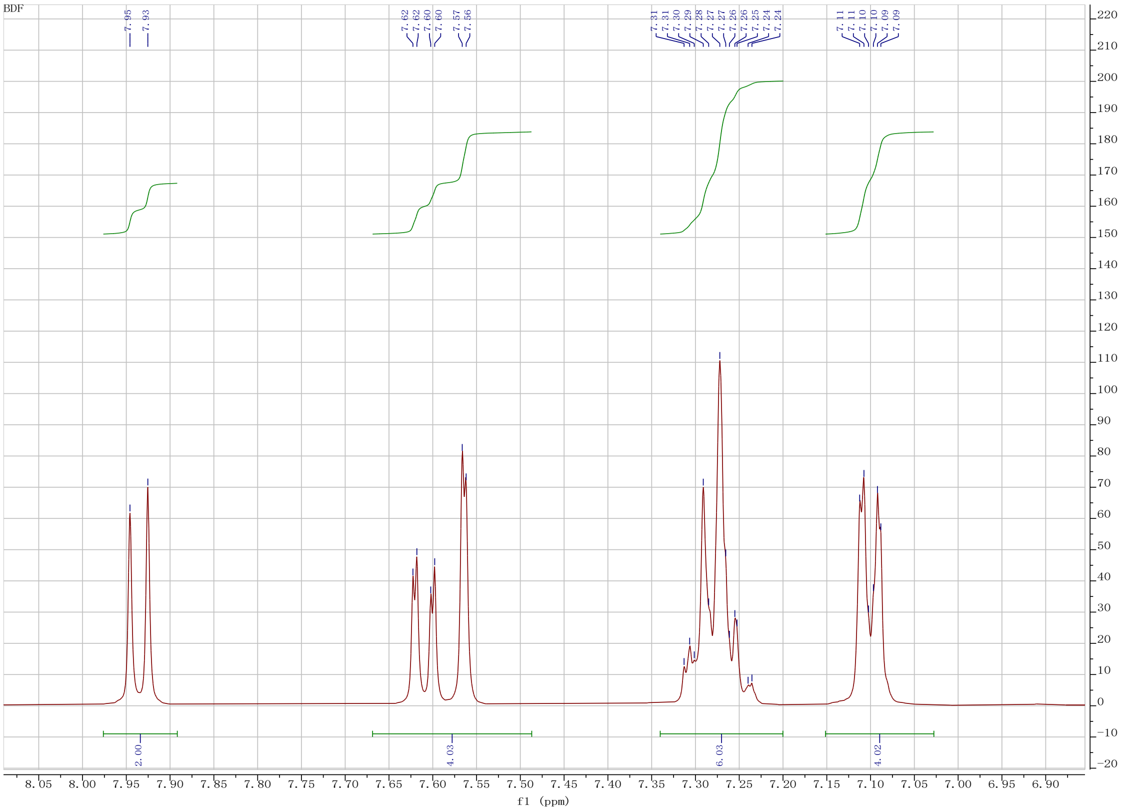

Fig. S3 ^{1}H NMR spectrum of DBDF, ^{1}H NMR (400 MHz, DMSO-d_6) δ 7.94 (d, J = 8.2 Hz,

2H), 7.67 – 7.49 (m, 4H), 7.34 – 7.20 (m, 6H), 7.15 – 7.03 (m, 4H).

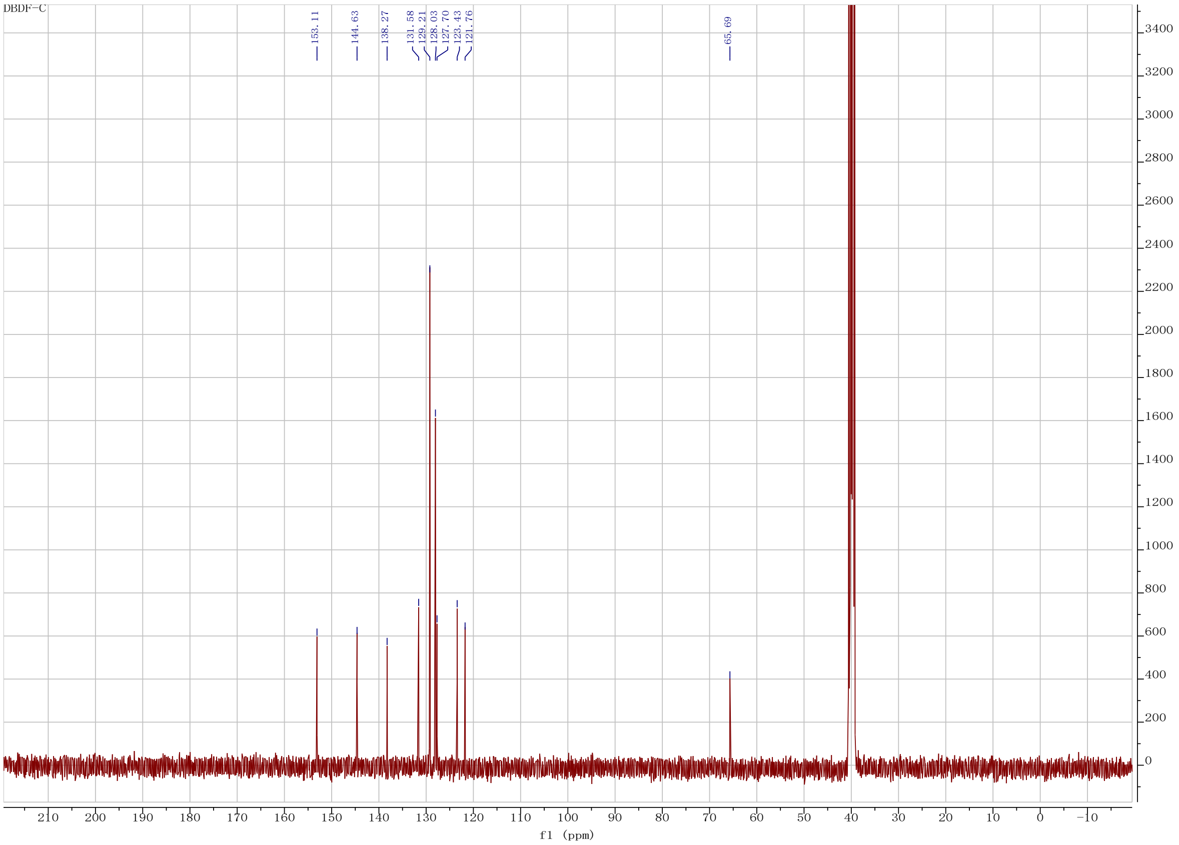

Fig. S4 ^{13}C NMR spectrum of DBDF， ^{13}C NMR (100 MHz, DMSO) δ 153.11, 144.63, 138.27, 131.58, 129.21, 128.03, 127.70, 123.43, 121.76, 65.69.

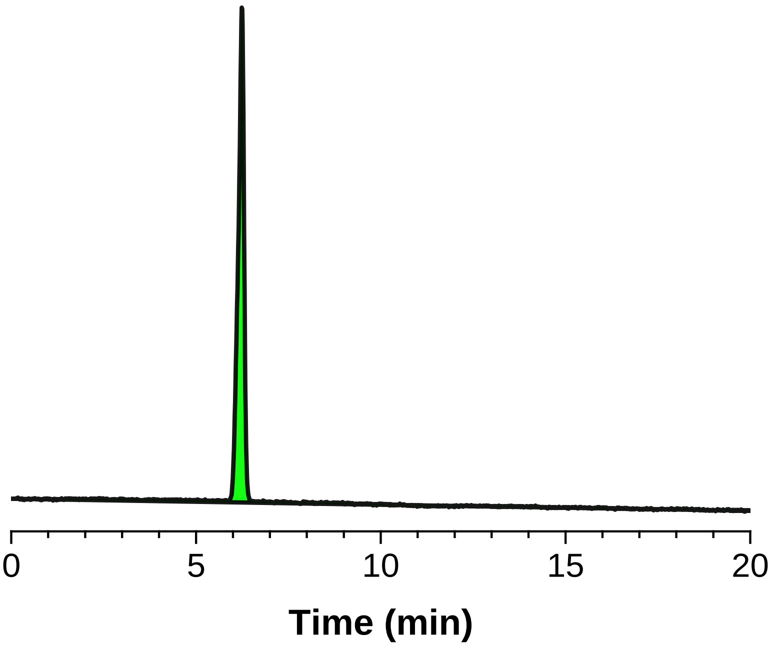

Figure S5 HPLC spectrum of BDF.

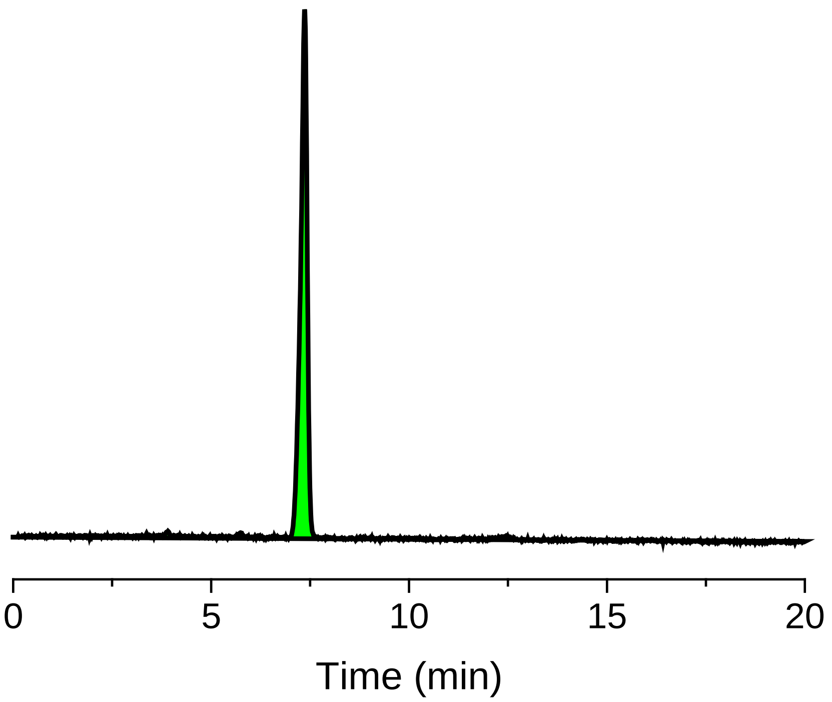

Figure S6 HPLC spectrum of DBDF.

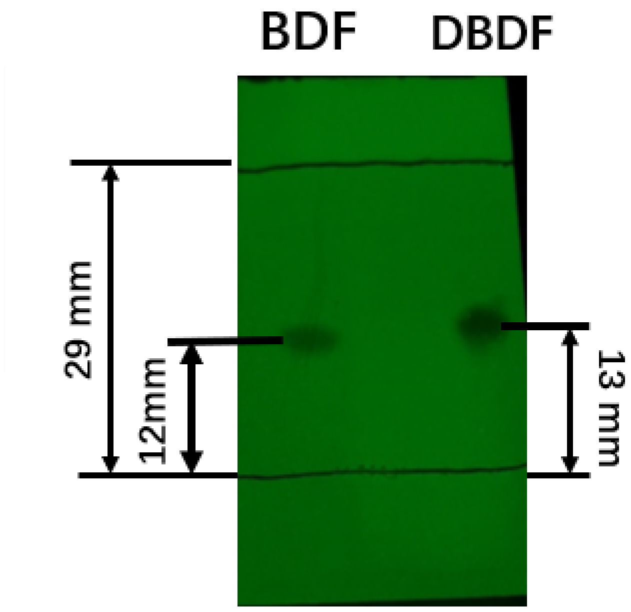

Figure S7 Photographs of isolated BDF and DBDF on thin-layer chromatography (TLC) plate under 254 nm UV light irradiation after being developed in 1/10 chloroform-petroleum mixture.

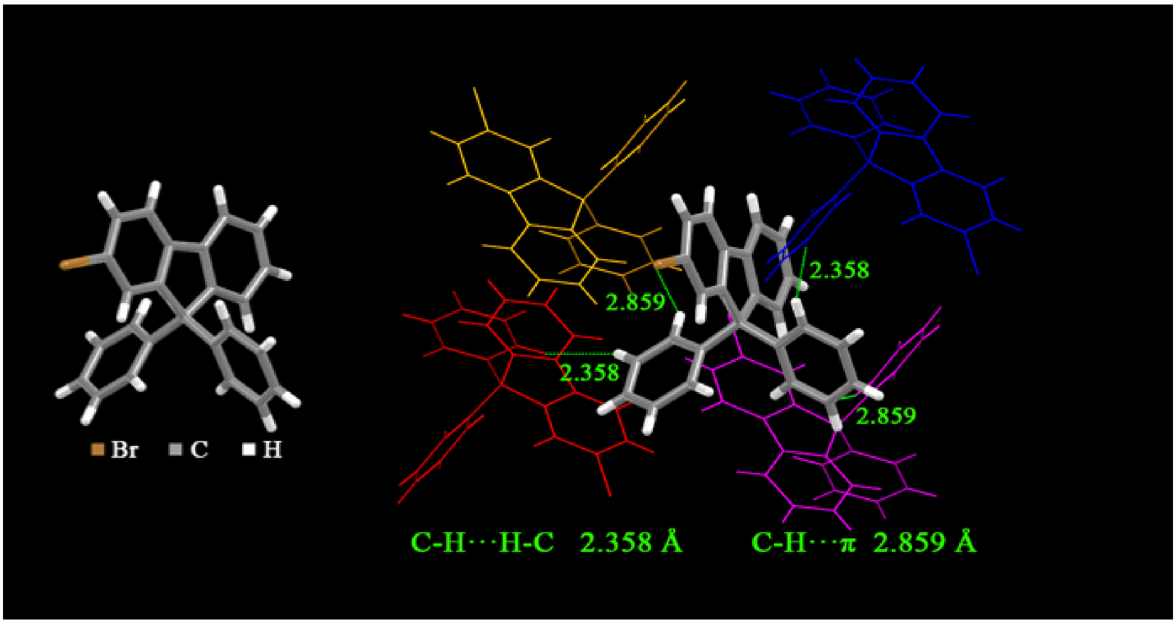

Figure S8 Single crystal structure and intermolecular interaction of BDF

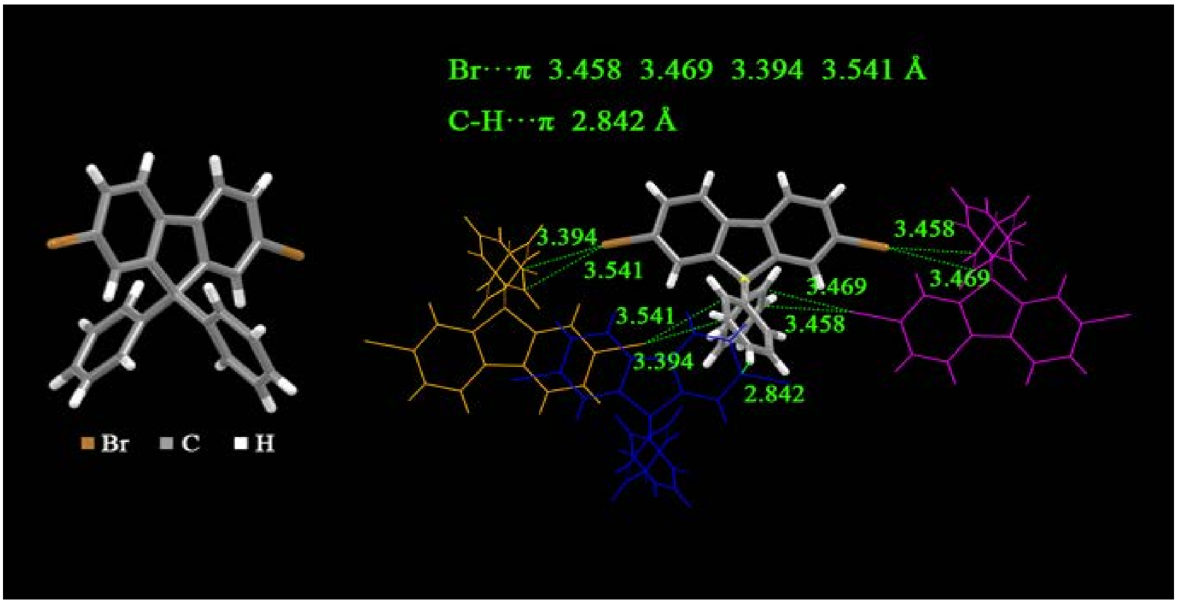

Figure S9 Single crystal structure and intermolecular interaction of DBDF.

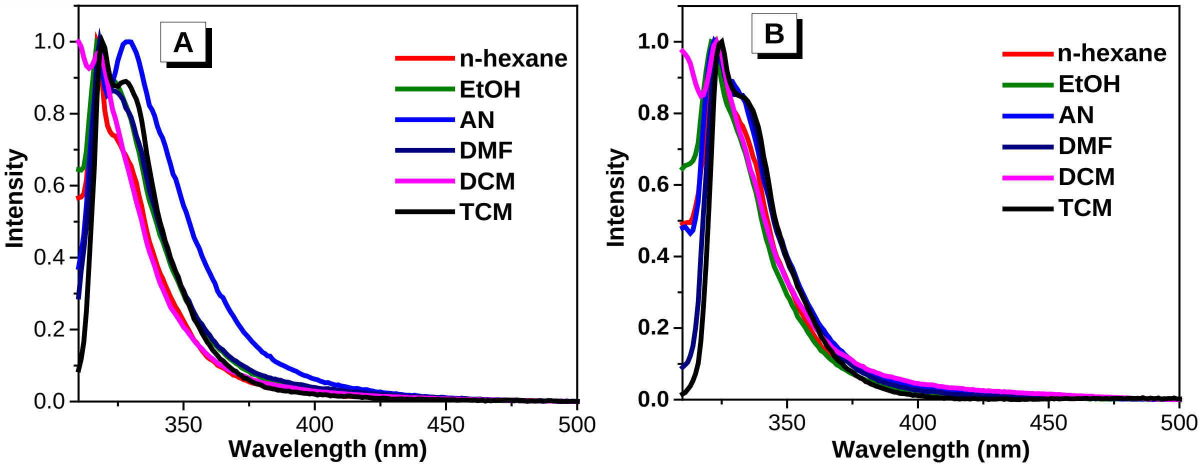

Figure S10 The prompt spectra of BDF (A) and DBDF (B) in different common solvents under air atmosphere, concentration=2.0×10^{-5} M, excitation wavelength =290 nm.

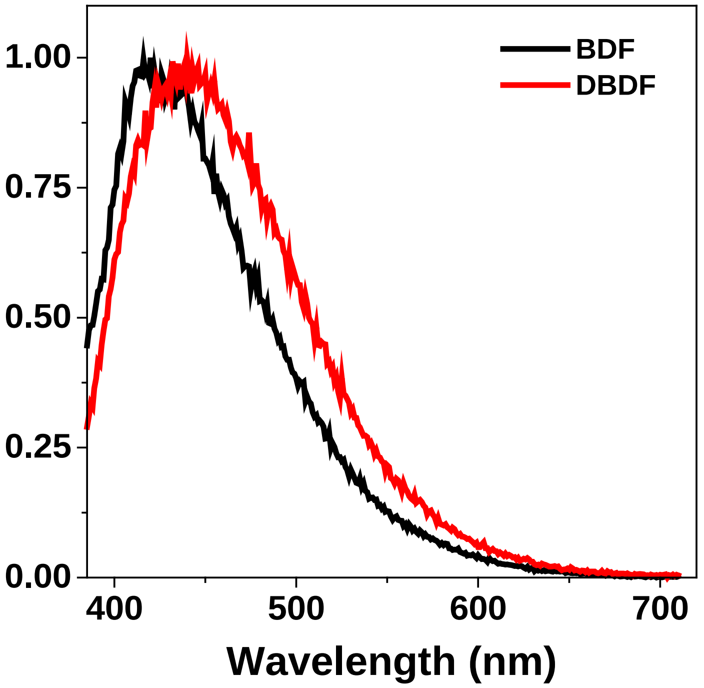

Figure S11 Delayed emission spectra of BDF and DBDF solutions in Ar bubbled TCM solvents. Concentration=2.0×10^{-5} M, delayed time =0.1 ms, Exaction wavelength =365 nm.

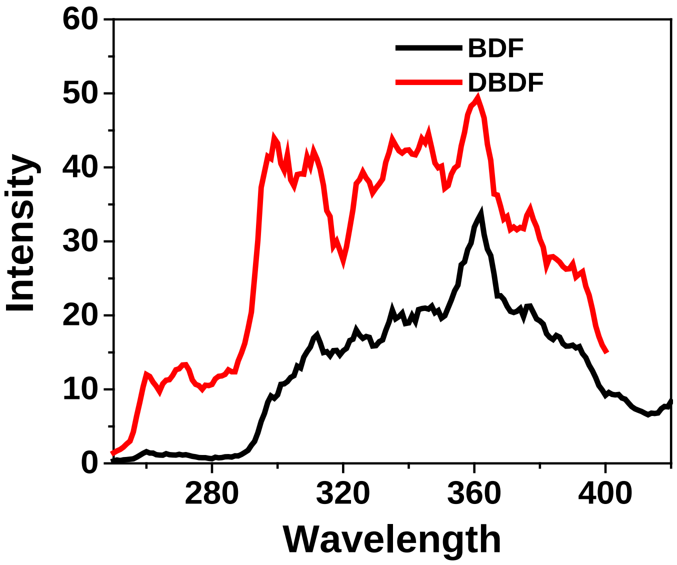

Figure S12 The excitation spectra of **BDF** and **DBDF** in chloroform solutions (2.0×10^{-5} M) at room temperature.

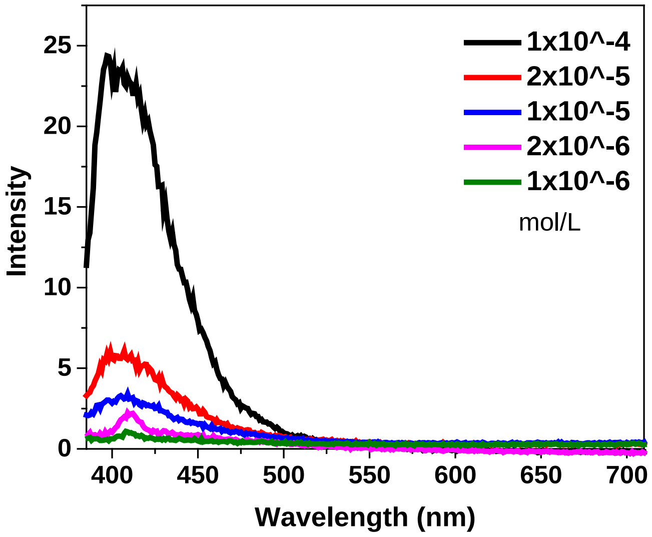

Figure S13 Delay emission spectra of BDF in Ar bubbled TCM solutions with different concentration, excitation wavelength =365 nm, delay time =0.1 ms.

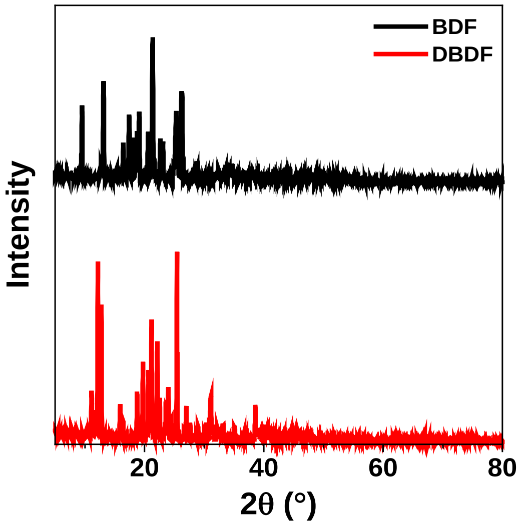

Figure S14 The XRD patterns of crystals of BDF and DBDF.

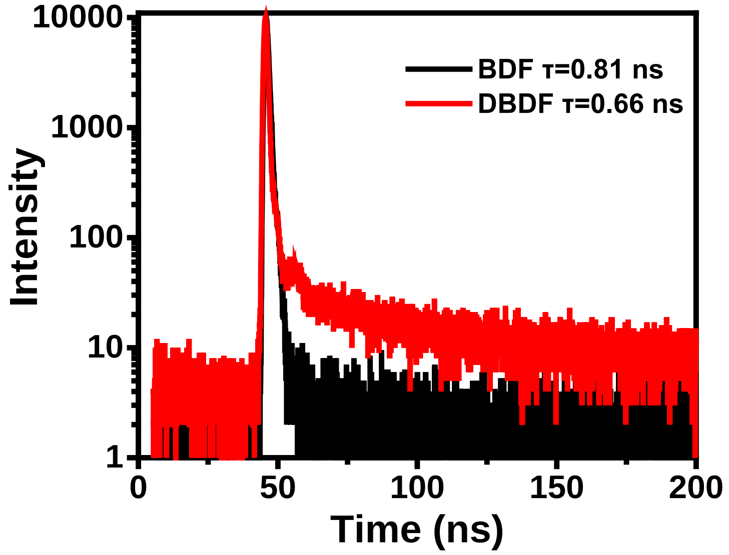

Figure S15 The fluorescent lifetime curves of crystalline powders of BDF and DBDF.

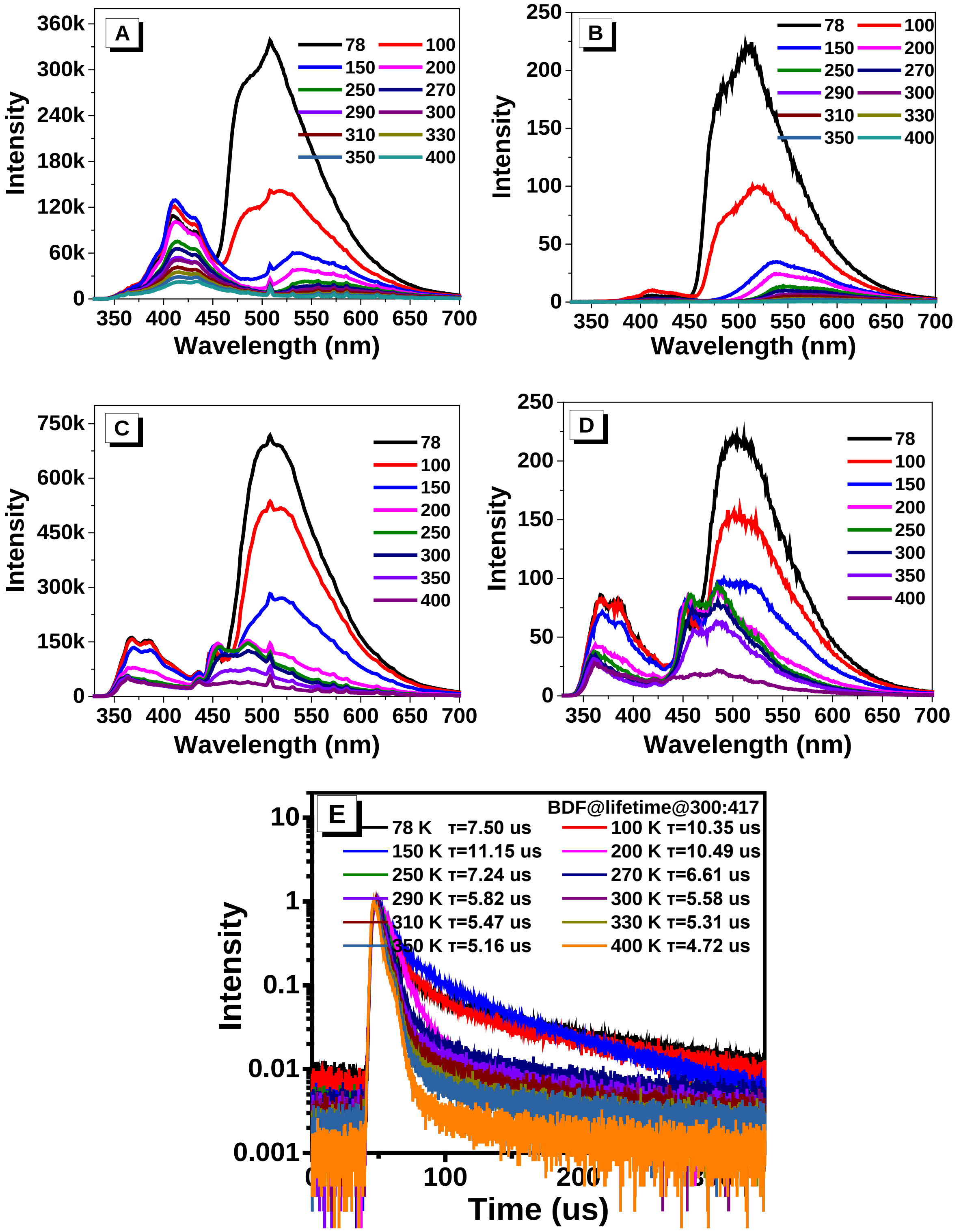

Figure S16 The prompt (A, C) and delay (B, D) emission spectra of BDF (A, B) and DBDF (C, D) at crystalline state under various temperatures (78 - 400 K), excitation wavelength = 300 nm, delay time = 0.1 ms; phosphorescence delay curves of crystalline powders in different temperature of BDF (E), excitation wavelength = 300 nm, monitored at 417 nm.

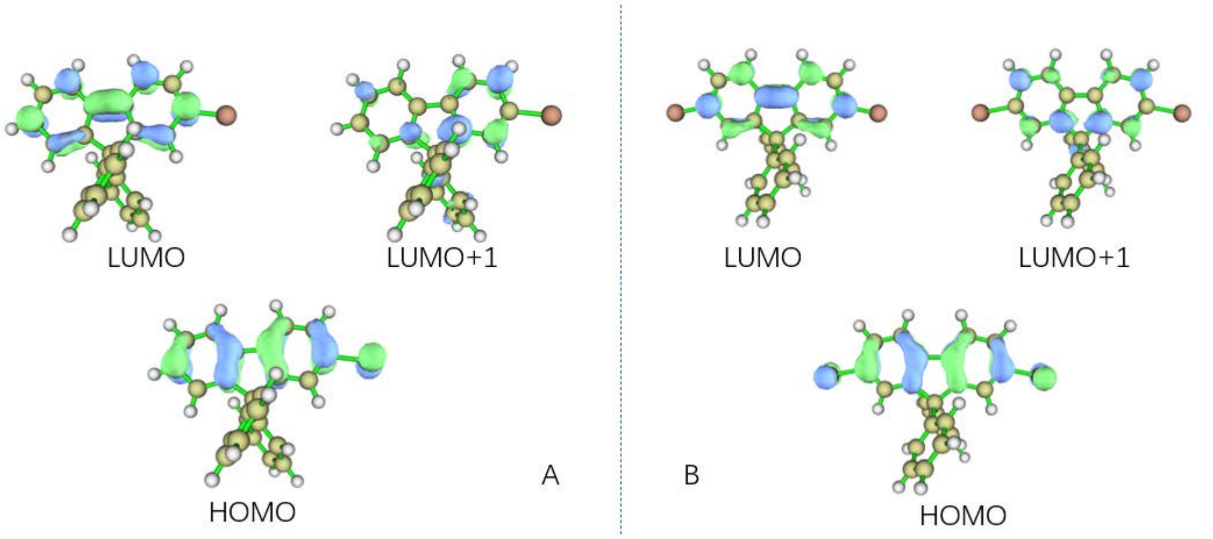

Figure S17 Simulated HOMO and LUMO distribution of BDF and DBDF calculated using the M06-2X/def-TZVP method.

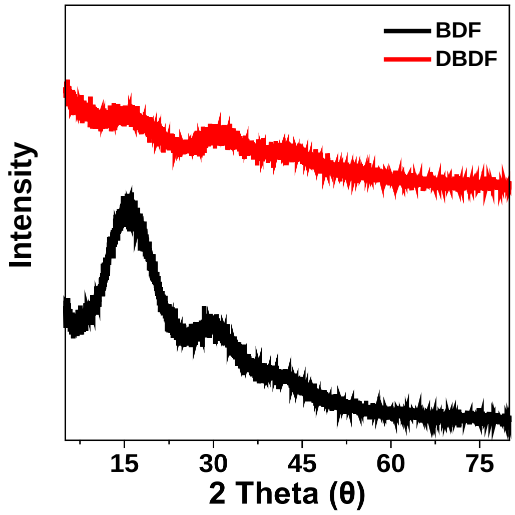

Figure S18 The XRD patterns of the films of **BDF** and **DBDF**

frontiers

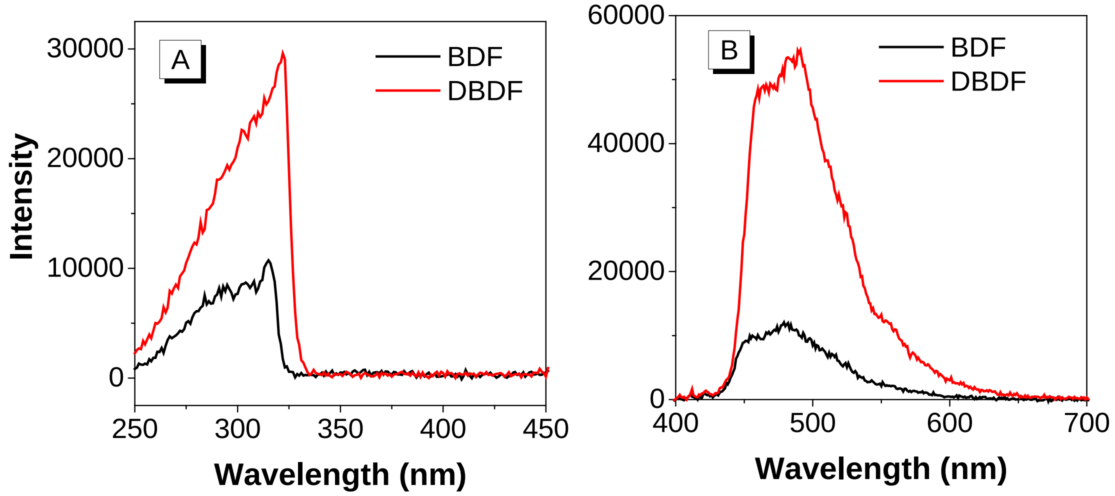

Figure S19 Excitation (A) and prompt (B) emission spectra of doped film.

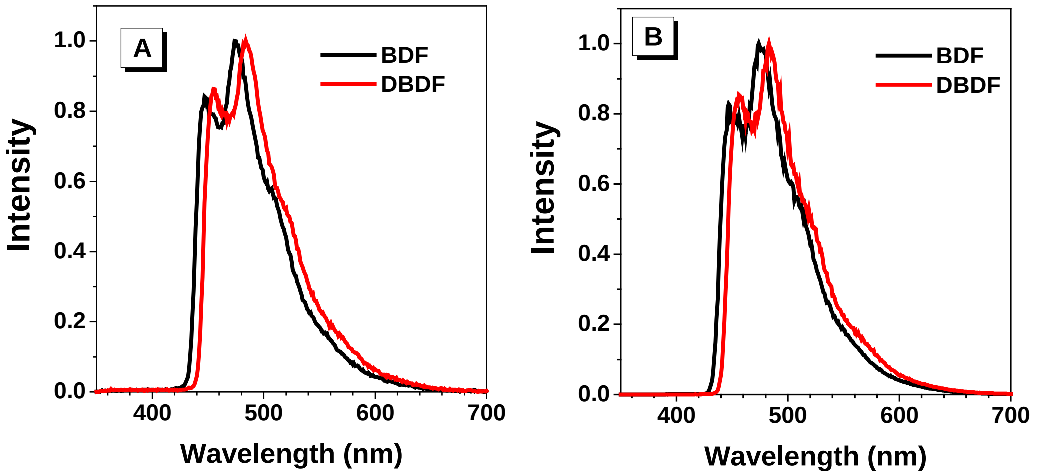

Figure S20 The prompt (A) and delay (B) emission spectra of BDF and DBDF doped film at 78 K, Delay time =1 ms, exaction wavelength = 300 nm.

Table S1 Crystal data and structure refinement for BDF and DBDF

Identification code	BDF	DBDF
Empirical formula	$C_{25}H_{17}Br$	$C_{25}H_{16}Br_2$
Formula weight	397.29	476.2
Temperature/K	293(2)	296.15
Crystal system	monoclinic	triclinic
Space group	$P2_1/c$	P-1
a/Å	12.234(3)	8.783(4)
b/Å	11.126(3)	8.950(2)
c/Å	17.740(3)	14.216(3)
α/°	90	91.14(2)
β/°	130.881(10)	99.82(2)
γ/°	90	117.38(2)
Volume/Å^3	1825.7(8)	971.7(5)
Z	4	2
ρ_{calc}g/cm^3	1.445	1.628
μ/mm^{-1}	2.256	4.178
F(000)	808	472
Crystal size/mm^3	0.31 × 0.27 × 0.2	0.26 × 0.21 × 0.17
Radiation	MoKα (λ = 0.71073)	MoKα (λ = 0.71073)
2Θ range for data collection/°	4.612 to 49.998	2.926 to 49.994
Index ranges	$-14 \le h \le 12$, $-12 \le k \le 13$, $-16 \le l \le 21$	$-10 \le h \le 10$, $-10 \le k \le 9$, $-16 \le l \le 16$
Reflections collected	8978	4888
Independent reflections	3203 [R_{int} = 0.0436, R_{sigma} = 0.0543]	3381 [R_{int} = 0.0328, R_{sigma} = 0.1551]
Data/restraints/parameters	3203/5/235	3381/0/244
Goodness-of-fit on F^2	1.094	1.078
Final R indexes [I>=2σ (I)]	R_1 = 0.0608, wR_2 = 0.1631	R_1 = 0.0621, wR_2 = 0.1772
Final R indexes [all data]	R_1 = 0.1040, wR_2 = 0.1770	R_1 = 0.1008, wR_2 = 0.2019
Largest diff. peak/hole / e Å^{-3}	1.38/-0.64	0.49/-0.70
CCDC No.	2109386	2109387

Table S2 Triplet excited states of BDF and DBDF containing the same orbital transition components of S1.

Excited State	Energy (eV)	Transition configuration (%)
BDF		
T_1	3.5016	H → L 78.4%
T_2	4.2194	H → L+1 61.4%, H-1 → L+2 7.0%, H → L 6.0%
T_3	4.2837	H-1 → L+2 11.4%, H-1 → L+1 8.8%, H-1 → L+4 8.5%, H-2 → L+2 5.6%, H-5 → L+2 5.5%, H-4 → L+3 5.1%
T_4	4.4377	H-5 → L+1 17.9%, H → L+8 6.9%, H-4 → L+1 5.9%, H-7 → L 5.8%, H-2 → L+6 5.6%
T_5	4.4377	H-5 → L+1 17.9%, H → L+8 6.9%, H-4 → L+1 5.9%, H-7 → L 5.8%, H-2 → L+6 5.6%
S_1	4.6503	H → L 71.3%, H → L+1 12.7%
DBDF		
T_1	3.4284	H → L 80.6%
T_2	4.1563	H → L+1 73.2%, H → L 6.0%
T_3	4.2803	H-1 → L+1 12.5%, H-2 → L+2 12.5%, H-1 → L+4 10.6%, H-4 → L+3 9.0%, H-4 → L+2 7.0%, H-1 → L+6 5.8%, H-3 → L+6 5.7%, H → L+2 5.1%
T_4	4.2854	H-1 → L+2 25.8%, H-3 → L+3 15.5%, H-4 → L+6 13.0%, H-2 → L+4 11.9%, H-1 → L+5 8.0%
T_5	4.4100	H-5 → L+1 26.0%, H-6 → L 11.3%, H → L+9 10.0%, H-2 → L+5 8.7%, H-7 → L+2 7.9%
S_1	4.5509	H → L 77.5%, H → L+1 9.4%

Table S3 Cartesian coordinates of optimized BDF in S_0 state

atom	x	y	z
C	2.770484	1.986501	-0.05108
C	2.805169	0.601448	0.060086
C	1.648489	-0.16684	0.075225
C	0.436051	0.491229	-0.01595
C	0.379825	1.882323	-0.13197
C	1.546362	2.6347	-0.15024
H	3.694628	2.547779	-0.06234
H	1.703572	-1.24467	0.157736
H	1.513151	3.713519	-0.24023
C	-1.82401	1.141029	-0.15903
C	-3.20244	1.239368	-0.21001
C	-3.78084	2.499482	-0.32963
C	-2.98742	3.642741	-0.38913
C	-1.60301	3.546422	-0.3294

C	-1.02759	2.287835	-0.21409
H	-3.8224	0.352161	-0.15923
H	-4.85842	2.593137	-0.37376
H	-3.45546	4.614752	-0.47903
H	-0.98751	4.436883	-0.37165
C	-0.96646	-0.11854	-0.02664
C	-1.32934	-0.8151	1.288021
C	-1.07463	-1.05166	-1.23677
C	-0.6625	-0.51974	2.473879
C	-2.4095	-1.69785	1.33141
C	-1.52801	-0.59033	-2.46954
C	-0.62662	-2.36996	-1.14024
C	-1.06139	-1.09708	3.673725
H	0.17376	0.167835	2.465401
C	-2.80854	-2.27319	2.528606
C	-1.53875	-1.42841	-3.57865
H	-1.87392	0.430844	-2.56792
C	-0.6361	-3.20591	-2.24686
C	-2.13377	-1.97592	3.706338
H	-0.52783	-0.85696	4.584767
H	-3.64643	-2.959	2.540557
C	-1.09445	-2.73783	-3.47233
H	-1.89706	-1.05202	-4.52859
H	-0.28705	-4.22638	-2.15069
H	-2.44145	-2.42808	4.640518
H	-1.10536	-3.39037	-4.33598
H	-2.93194	-1.94872	0.415824
H	-0.28236	-2.74946	-0.18534
Br	4.493885	-0.26942	0.192444

Table S4 Cartesian coordinates of optimized DBDF in S_0 state

atom	x	y	z
C	-3.00153	-2.53973	0.049962
C	-3.42345	-1.21524	0.058947
C	-2.5265	-0.15508	0.043116
C	-1.17672	-0.45282	0.0257
C	-0.73298	-1.7774	0.012297
C	-1.64308	-2.82569	0.024306
H	-3.7327	-3.33618	0.059665
H	-2.88119	0.867484	0.045455
H	-1.30899	-3.85585	0.013603
C	1.176706	-0.45282	-0.02563
C	2.526484	-0.15508	-0.04314
C	3.42343	-1.21524	-0.05893
C	3.001517	-2.53973	-0.04983
C	1.64307	-2.82569	-0.02411

C	0.732968	-1.7774	-0.01214
H	2.88118	0.867481	-0.04556
H	3.732685	-3.33618	-0.05951
H	1.308977	-3.85585	-0.01334
C	-8E-06	0.523715	0.000038
C	0.105849	1.372694	1.270615
C	-0.10585	1.372648	-1.27057
C	-0.50165	0.978135	2.459322
C	0.901196	2.51948	1.274284
C	0.502029	0.978227	-2.45912
C	-0.90155	2.519188	-1.27445
C	-0.32356	1.715894	3.623901
H	-1.11756	0.088039	2.481496
C	1.080275	3.254647	2.436496
C	0.323977	1.715893	-3.62376
H	1.118217	0.088318	-2.48112
C	-1.0806	3.254256	-2.43673
C	0.46614	2.855817	3.617409
H	-0.80692	1.39507	4.538054
H	1.698661	4.143242	2.418442
C	-0.46608	2.855575	-3.61749
H	0.807638	1.395184	-4.5378
H	-1.69927	4.142658	-2.41885
H	0.601991	3.431292	4.524101
H	-0.6019	3.430977	-4.52423
H	1.370838	2.848258	0.354712
H	-1.37152	2.847841	-0.355
Br	-5.29038	-0.84382	0.088747
Br	5.290361	-0.84383	-0.08884
